# Night shifts, insomnia, anxiety, and depression among Chinese nurses during the COVID-19 pandemic remission period: A network approach

**DOI:** 10.3389/fpubh.2022.1040298

**Published:** 2022-12-05

**Authors:** Pu Peng, Mining Liang, Qian Wang, Lulu Lu, Qiuxia Wu, Qiongni Chen

**Affiliations:** ^1^Clinical Nursing Teaching and Research Section, The Second Xiangya Hospital, Central South University, Changsha, China; ^2^Department of Psychiatry, National Clinical Research Center for Mental Disorders, The Second Xiangya Hospital of Central South University, Changsha, Hunan, China

**Keywords:** COVID-19 pandemic, depression, anxiety, insomnia, network analysis

## Abstract

**Background:**

The outbreak of the COVID-19 pandemic imposed a heavy workload on nurses with more frequent night shifts, which led to higher levels of insomnia, depression, and anxiety among nurses. The study aimed to describe the symptom-symptom interaction of depression, anxiety, and insomnia among nurses and to evaluate the impact of night shifts on mental distress *via* a network model.

**Methods:**

We recruited 4,188 nurses from six hospitals in December 2020. We used the Insomnia Severity Index, Patient Health Questionnaire-9, and Generalized Anxiety Disorder Scale-7 to assess insomnia, depression, and anxiety, respectively. We used the gaussian graphical model to estimate the network. Index expected influence and bridge expected influence was adapted to identify the central and bridge symptoms within the network. We assessed the impact of night shifts on mental distress and compared the network structure based on COVID-19 frontline experience.

**Results:**

The prevalence of depression, anxiety, and insomnia was 59, 46, and 55%, respectively. Nurses with night shifts were at a higher risk for the three mental disorders. “Sleep maintenance” was the central symptom. “Fatigue,” “Motor,” “Restlessness,” and “Feeling afraid” were bridge symptoms. Night shifts were strongly associated with sleep onset trouble. COVID-19 frontline experience did not affect the network structure.

**Conclusion:**

“Sleep maintenance,” “Fatigue,” “Motor,” and “Restlessness” were important in maintaining the symptom network of anxiety, depression, and insomnia in nurses. Further interventions should prioritize these symptoms.

## Introduction

The COVID-19 pandemic has deeply enlarged the workload and worsened the mental state of healthcare workers (1–3); approximately 40% have experienced anxiety, depression, and insomnia symptoms during the pandemic (1, 2, 4). One of the major risk factors for increased mental problems might be the heavy workload, especially frequent night shifts. Studies consistently reported that night shifts were associated with a higher risk of burnout, sleep impairment, depression, anxiety, and low quality of life among healthcare workers (5–10).

Network analysis is an emerging and promising tool for understanding the psychopathology of mental disorders (11). It assumes that symptoms are components rather than the reflection of mental disorders (12). The network model allows relationships to be identified within symptoms to find the “central symptom” considered to have the strongest influence on the other symptoms in the network (13, 14). It also provides an opportunity to establish comorbidity at the symptom level by identifying “bridge symptoms” (15). Central and bridge symptoms are pivotal in developing and maintaining mental disorders (15, 16). Targeting these symptoms is of great clinical value.

Increasingly, studies use network analysis to describe the symptom network of anxiety and/or depression among different populations including adolescents, college students, the general population, and quarantined individuals during the pandemic (17–21). However, despite the high prevalence of psychological symptoms among nurses, there was no previous study describing the potential symptom-symptom interaction among the nursing population. Moreover, studies regarding the mental health of nurses were mainly carried out during the peak of the pandemic. Nevertheless, studies suggested the persistence of mental symptoms even long after the pandemic's initial peak (22–26). Describing the prevalence and network structure of depression, anxiety, and insomnia symptoms among nurses in the late stage of the pandemic would provide valuable insights into the long-term investigation, identification, and intervention for these symptoms in the nursing population.

Hence, we conducted the present study to assess the network structure of anxiety, depression, and insomnia symptoms in a large sample of Chinese nurses during the pandemic's remission period. We aimed to identify the central and bridge symptoms within this network. In particular, we examined the impact of night shifts and COVID-19 frontline experience on nurses' mental health.

## Methods

This study was performed based on the reporting standards for psychological network analyses of cross-sectional data (27).

### Study setting and participants

We conducted a secondary analysis using the data from our previous study (28). The study used a web-based questionnaire and was conducted in December 2020 in Hunan province, China, which had entered a remission period during the COVID-19 pandemic (8 months without any new local cases). Snowball sampling was used to recruit nurses from six local hospitals. All practicing nurses willing to participate in the survey were eligible. Student nurses or nurses on sick leave or maternity leave during 2020 were excluded. After participants provided informed consent, the questionnaire was distributed *via* an online survey platform (www.wjx.cn) and WeChat. Only participants who responded to all questions could submit the questionnaire, and we used identification numbers to avoid repeat submissions. Participation was voluntary with no compensation. The questionnaire took average 5–8 min to complete. Participants who took too short (< 2 min) or too long (>60 min) to complete the survey were excluded.

### Measures

We collected demographic (age, gender, education level, partnership status, family income) and work-related (work duration, night shifts, title, COVID-19 frontline experience, hospital level) characteristics *via* self-designed questionnaires. Nurses aiding Wuhan or working in local isolation wards during the pandemic were identified as frontline nurses. Chinese nurses worked in three shift schedules including the day shift (8:00 am−4:00 pm), evening shift (4:00 pm−0:00 am), and night shift (0:00 am−8:00 am). Night shifts were questioned through the single item “How many night shifts do you have per month?.”

To evaluate insomnia, we used the Chinese version of the Insomnia Severity Index (ISI), a validated questionnaire in both clinical and non-clinical populations (29, 30). It contains 7 items on severity of sleep disturbances and associated daytime symptoms, assessed on a five-point Likert scale, ranging from 0 (“not at all”) to 4 (“very serious”). Higher ISI scores indicate greater insomnia severity. ISI scores above 7 were used to identify potential insomnia.

Depression and anxiety symptoms were assessed with the 9-item Patient Health Questionnaire (PHQ-9) and 7-item General Anxiety Disorder scale (GAD-7), respectively, which are validated and widely used in Chinese populations (31, 32). Both questionnaires use four-point Likert scales to assess symptom frequency, from 0 (“not at all”) to 3 (“nearly every day”). Following previous studies (33, 34), a cutoff score of 5 was used to screen for depression and anxiety symptoms. We removed the item “trouble falling or staying asleep, or sleeping too much” (PHQ3), as it focused on sleep problems and could overlap with insomnia in the network analysis.

### Statistical analysis

All statistical analyses were conducted using R (ver. 4.2.0). We described continuous variables as the median and interquartile range (IRQ; 25–75%). Categorical data were presented as frequency and percentages. All tests were 2-tailed; *p* < 0.05 indicated statistical significance.

### Network estimation

We used the “describe” function in the R package “psych” to calculate means, standard deviations (SD), kurtosis, and skewness for PHQ-9, GAD-7, and ISI items. Items with an SD 2.5 times lower than the mean for all scale items were considered to be less informative and excluded. The “goldbricker” function in the R package “network tools” was used to identify redundant items.

Following previous research, we used the R packages “bootnet” and “qgraph” to estimate and visualize the network analysis (35). To estimate the network, we used the gaussian graphical model with the default of the EBICglasso model, which was widely used in psychological network models (36). The network model defined the symptom as a “node.” The “edge” between two nodes represents a unique association between two symptoms after controlling for all other variables in the network. Thicker edges indicate stronger associations (27). Red edges indicated a negative association while blue edges suggested a positive association.

To identify the network's central symptoms, we calculated the centrality indices “strength,” “betweenness,” “expected influence” (EI), and “closeness” *via* the R package “qgraph.” The EI index was chosen to quantify the importance of the node (37). We used the R package “MGM” to assess node predictability in the network. Predictability suggests the extent to which a node's variance can be explained by its neighbors (19). High predictability suggests that a symptom could be controlled by changing neighboring nodes. We assessed Spearman's rank-order correlations between mean item scores and both node strength and predictability, following previous studies (38–40).

To identify possible bridge symptoms linking the three mental symptoms, we used the R package “networktool” (15). We assessed the bridge expected influence (BEI) index, with a higher EI suggesting a stronger association with symptoms in other communities. Bridge symptoms were chosen with an 80th percentile BEI threshold (41).

To evaluate the impact of night shifts on depression, anxiety, and insomnia symptoms, we added night shifts to the network and used the “flow” function in Rpackage “qgraph.”

### Network stability and accuracy

To test the accuracy of edge estimations, we used non-parametric bootstrapping with 1000 bootstrap samples *via* the “bootnet” packages. We also tested bridge and center strength stability using a case-dropping bootstrap procedure (35). The correlation stability coefficient (CS-C) represented network stability. A CS-C higher than 0.5 was considered good.

### Network comparison

We compared the insomnia–depression–anxiety network based on COVID-19 frontline experience *via* the R package “Network Comparison tool.” The global network strength (absolute sum of all edge weights) and structure (distribution of edge weights) between both networks were evaluated.

### Ethical considerations

The study was approved by the ethics committee of the Second Xiangya Hospital of Central South University.

## Results

### Descriptive statistics

In total, 4,237 nurses participated in the survey and 4,188 validated responses were included in the final analysis (Table 1). The median age was 30 (26–35), and the median for years practicing was 8 (4–13). One-fifth of the participants worked as frontline nurses during the pandemic's peak. Most participants were women (98%), had a bachelor's degree (69%), had a junior title (61%), and were married (68%). Approximately half experienced depression (59.6%), anxiety (47%), and insomnia (55.5%) symptoms. 2917 (70%) of the nurses had at least one mental symptom. The median night shift frequency per month was 2 (1–3). 3153 nurses had at least one night shift per month. Compared with those without night shifts, nurses with night shifts were at a higher risk for depression (62 vs. 50%), anxiety (48 vs. 42%), and insomnia (57 vs. 48%) (all *p* < 0.001).

**Table 1:** Characteristics of participants.

**Variable**	**Overall,** ***N* = 4,188^a^**	**Nurses without shifts,** ***N* = 1,035^a^**	**Nurses with shifts,** ***N* = 3,153^a^**	***p*-value^b^**
**Workplace**				< 0.001
Tertiary hospital	1,290 (31%)	214 (21%)	1,076 (34%)	
Secondary hospital	898 (21%)	281 (27%)	617 (20%)	
Primary hospital	2,000 (48%)	540 (52%)	1,460 (46%)	
**Gender**				< 0.001
Female	4,099 (98%)	1,027 (99%)	3,072 (97%)	
Male	89 (2%)	8 (1%)	81 (3%)	
**Age, years**	30 (26, 35)	36 (30, 43)	29 (25, 32)	< 0.001
**Practicing years**	8 (4, 13)	15 (9, 23)	7 (3, 10)	< 0.001
**Title**				< 0.001
Junior title	2,536 (61%)	340 (33%)	2,196 (70%)	
Nurses in charge	1,464 (35%)	537 (52%)	927 (29%)	
Chief nurses	188 (5%)	158 (15%)	30 (1%)	
**Head nurse**	370 (9%)	260 (25%)	110 (4%)	< 0.001
**Education**				0.2
Junior college or below	1,110 (27%)	254 (25%)	856 (27%)	
Bachelor degree	2,910 (69%)	733 (71%)	2,177 (69%)	
Master degree or above	168 (4%)	48 (4%)	120 (4%)	
**Partnership**				< 0.001
Single	840 (20%)	58 (5%)	782 (25%)	
Partnered	382 (9%)	18 (2%)	364 (11%)	
Married	2,866 (68%)	920 (89%)	1,946 (62%)	
Widowed	100 (3%)	39 (4%)	61 (2%)	
**Family monthly income, CNY**				< 0.001
< 10,000	2,063 (50%)	452 (44%)	1,611 (51%)	
10,000–30,000	1,684 (40%)	479 (46%)	1,205 (38%)	
30,000–50,000	221 (5%)	49 (5%)	172 (6%)	
>50,000	220 (5%)	55 (5%)	165 (5%)	
**Frontline experience**	882 (21%)	184 (18%)	698 (22%)	0.003
GAD7	4 (1, 7)	3 (0, 7)	4 (1, 7)	< 0.001
PHQ9	6 (2, 9)	4 (2, 8)	6 (3, 9)	< 0.001
ISI	8 (4, 11)	7 (3, 11)	8 (4, 12)	< 0.001
Anxiety	1,935 (46%)	431 (42%)	1,504 (48%)	< 0.001
Depression	2,457 (59%)	515 (50%)	1,942 (62%)	< 0.001
Insomnia	2,287 (55%)	501 (48%)	1,786 (57%)	< 0.001
At least one distress	2,917 (70%)	669 (65%)	2,248 (71%)	< 0.001

^a^Median (IQR); n (%).

^b^Wilcoxon rank sum test; Pearson's Chi-squared test.

### Insomnia–depression–anxiety network structure

No items were excluded for low item informativeness or redundancy. Table 2 presents the means and SDs for all items.

**Table 2:** Descriptive statistics of the items in the insomnia-depression-anxiety network.

**Items**	**Item content**	**Mean**	**SD**	**Strength**	**Predictability**
PHQ1	Anhedonia	0.86	0.68	0.25	0.64
PHQ2	Sad mood	0.75	0.66	0.29	0.64
PHQ4	Fatigue	0.95	0.73	0.51	0.64
PHQ5	Appetite	0.70	0.72	−1.29	0.46
PHQ6	Worthless	0.61	0.71	0.37	0.60
PHQ7	Concentration	0.57	0.72	−1.07	0.49
PHQ8	Motor	0.39	0.61	0.83	0.58
PHQ9	Death	0.23	0.51	−1.09	0.45
GAD1	Nervous	0.73	0.68	0.21	0.68
GAD2	Uncontrollable worry	0.57	0.70	0.69	0.72
GAD3	Excessive worry	0.77	0.74	0.69	0.71
GAD4	Trouble relaxing	0.69	0.74	0.77	0.72
GAD5	Restlessness	0.42	0.61	0.47	0.65
GAD6	Irritability	0.79	0.74	0.17	0.65
GAD7	Feeling afraid	0.47	0.66	0.65	0.66
ISI1	Sleep onset	0.91	0.98	0.28	0.64
ISI2	Sleep maintenance	0.97	1.05	1.67	0.72
ISI3	Early wakening	0.82	0.99	−1.21	0.57
ISI4	Sleep dissatisfaction	1.98	1.03	0.83	0.57
ISI5	Daytime disfunction	1.59	1.03	−1.57	0.35
ISI6	Noticeability	0.86	1.01	−2.46	0.18
ISI7	Sleep induced distress	1.24	1.13	0.00	0.50

**Figure 1:**
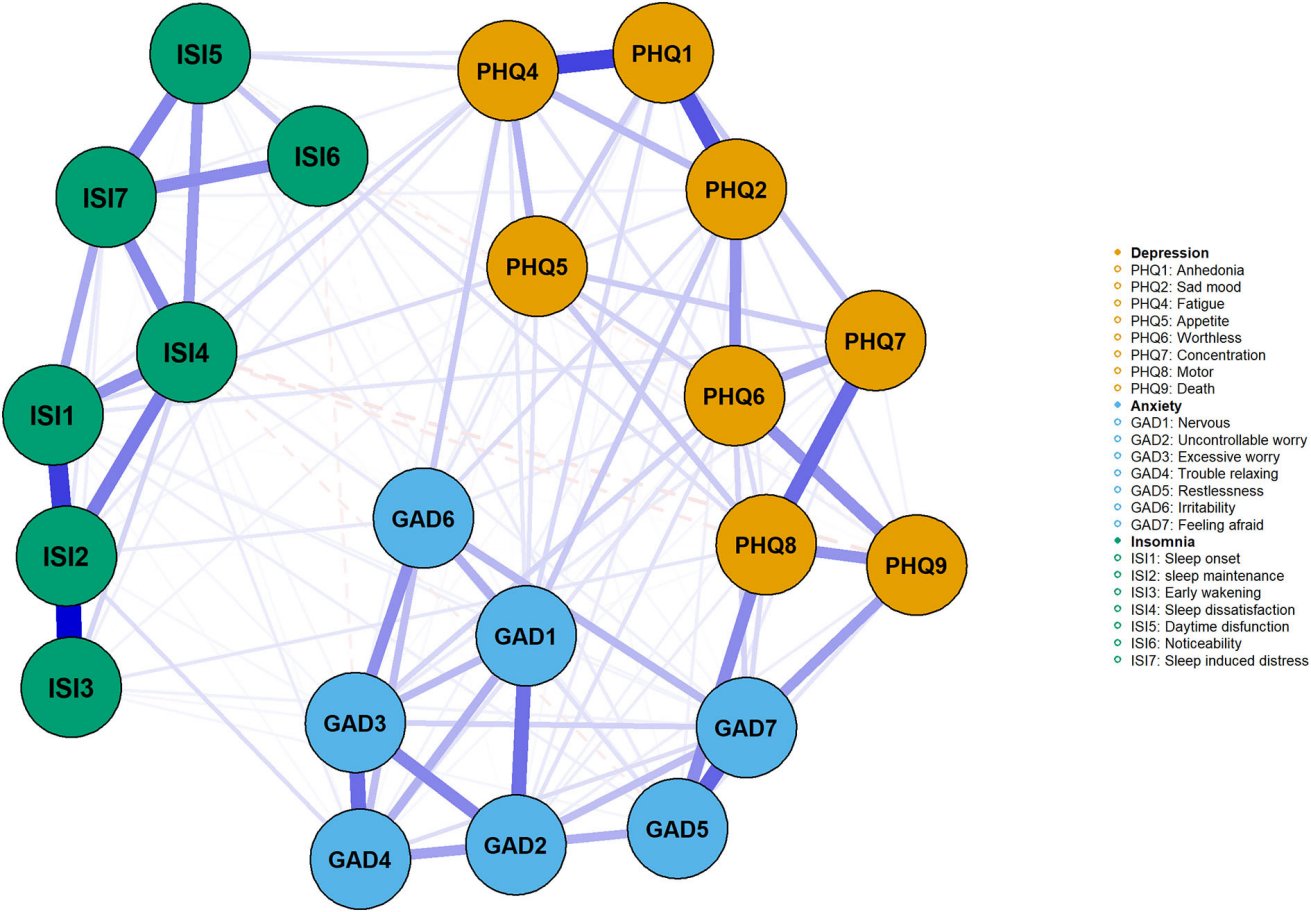
The network of insomnia, depression, anxiety symptoms in nurses. The green, blue, and orange nodes represented insomnia, anxiety, and depression symptoms, respectively. The blue edges represented positive association, while the red represented the negative association. Thicker edges suggested stronger association.

Figure 1 shows insomnia–depression–anxiety network, which had a density of 0.68 (156/231 edges) and a mean weight of 0.043. Supplementary Table S1 shows the correlation matrices. The nodes' mean predictability was 0.581, suggesting that 58% of a node's variance could be explained by adjacent nodes. ISI6 (noticeability) had the lowest predictability at 0.18, while GAD2 (uncontrollable worry), GAD4 (trouble relaxing), and ISI2 (sleep maintenance) had the highest predictability (0.72). We found no relationship between node predictability and mean value.

Following previous research (39), insomnia symptoms were divided into sleep disturbance (ISI1, ISI2, ISI3) and daytime dysfunction (ISI5, ISI6, ISI7) groups. ISI4 (sleep dissatisfaction) was connected to both groups. The strongest edge within the insomnia symptom network was ISI2 (sleep maintenance)–ISI3 (early wakening), which was also the strongest edge in the network according to the edge-differ test (Supplementary Figure S1). ISI2 (sleep maintenance)–ISI1 (sleep onset) was the second strongest edge. Within the depression symptom communities, the strongest edge was PHQ1 (anhedonia)–PHQ4 (fatigue), followed by PHQ1 (anhedonia)–PHQ2 (sad mood). The most robust edges within anxiety symptom communities were GAD7 (feeling afraid)–GAD5 (restlessness) and GAD3 (excessive worry)–GAD4 (trouble relaxing). The most robust transdiagnostic edge within the network was GAD5 (restlessness)–PHQ8 (motor).

### Central and bridge symptoms

**Figure 2:**
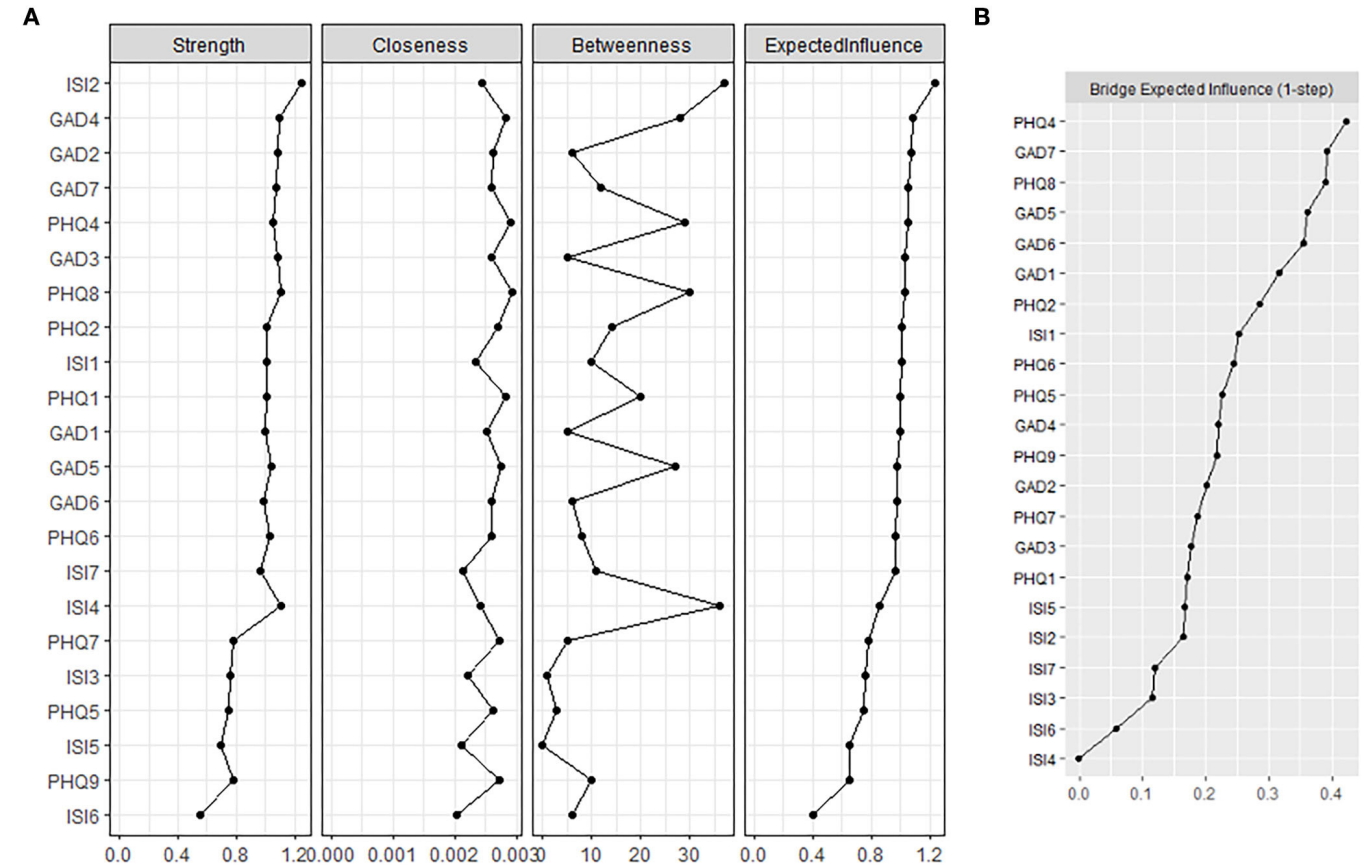
Centrality index and bridge expected influence of the nodes. **(A)** Centrality index of the nodes within the network. Higher expected influence suggested being more influential. **(B)** Bridge expected influence of the nodes. Nodes with higher bridge expected influence were considered to be the bridge symptoms which drove the comorbidity.

The centrality indices strength, closeness, EI, and betweenness are presented in Supplementary Table S2. The centrality index (Figure 2A) revealed the most central symptom of the insomnia–depression–anxiety network was ISI2 (sleep maintenance), which was statistically stronger than other symptoms (Supplementary Figure S1). Other central symptoms included GAD4 (trouble relaxing), GAD2 (uncontrollable worry), and GAD7 (feeling afraid). PHQ4 (fatigue) was the most central symptom in depression communities. We found no association between node strength with the mean value.

PHQ4 (fatigue), GAD7 (feeling afraid), PHQ8 (motor), and GAD5 (restlessness) held the highest BEI (Figure 2B), suggesting they served as bridge symptoms in the insomnia–depression–anxiety network. ISI1 (sleep onset) showed a stronger connection with anxiety and depression symptom communities than other insomnia symptoms.

### Network stability and accuracy

**Figure 3:**
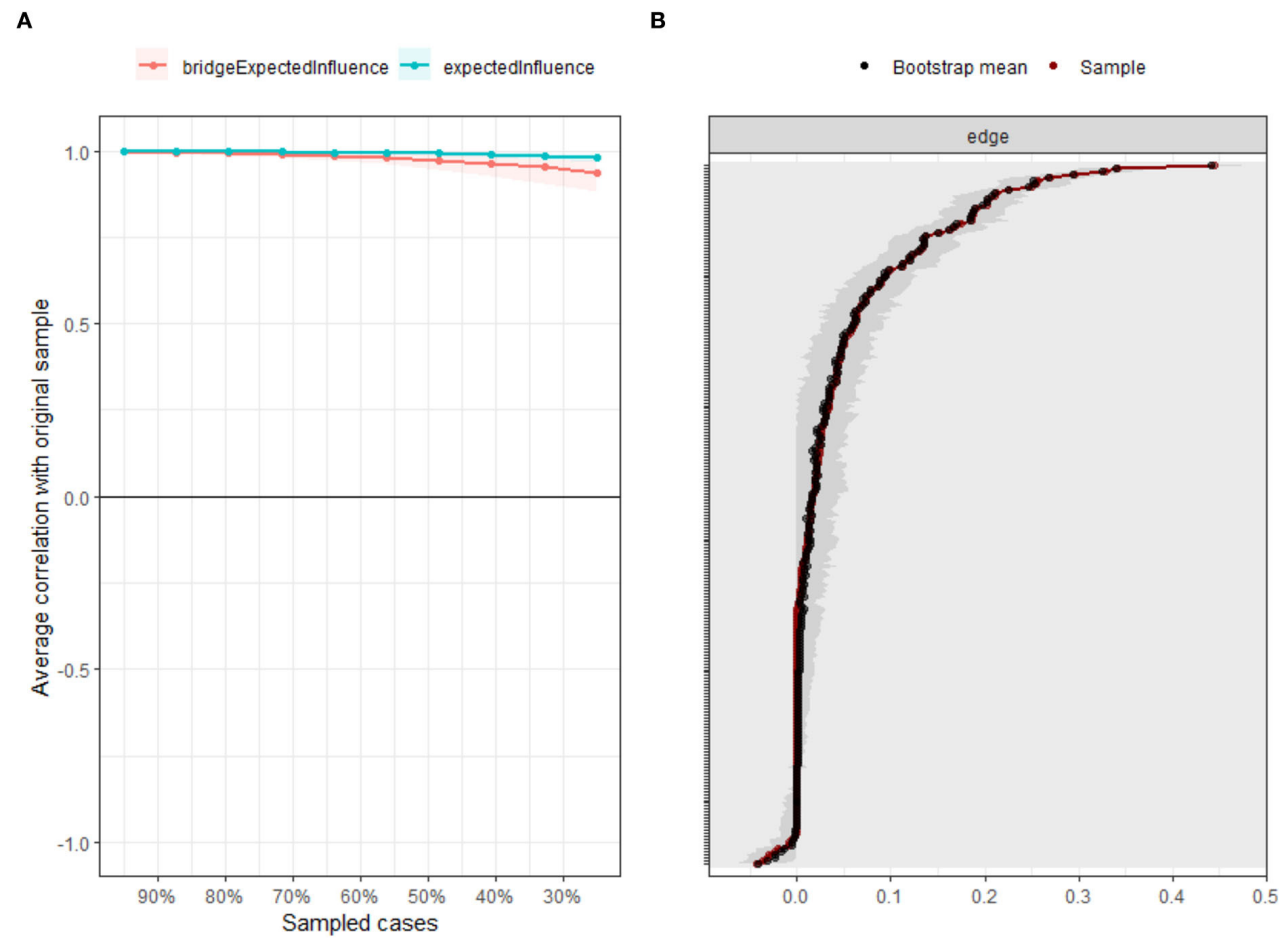
Accuracy and stability of the network. **(A)** The stability of central and bridge expected influence by case-dropping bootstrap. **(B)** The accuracy of the network edges by non-parametric bootstrapping.

Insomnia–depression–anxiety network exhibited excellent stability and accuracy. The case-dropping procedure found the CS-C of node and bridge expected influence was 0.75, indicating the network retained a correlation of 0.7 with the original data with 95% certainty even after omitting 75% of the raw data (Figure 3A). The bootstrapped 95% CIs were narrow, indicating the network's high accuracy (Figure 3B).

### Impact of night shifts on depression, anxiety, and insomnia symptoms

**Figure 4:**
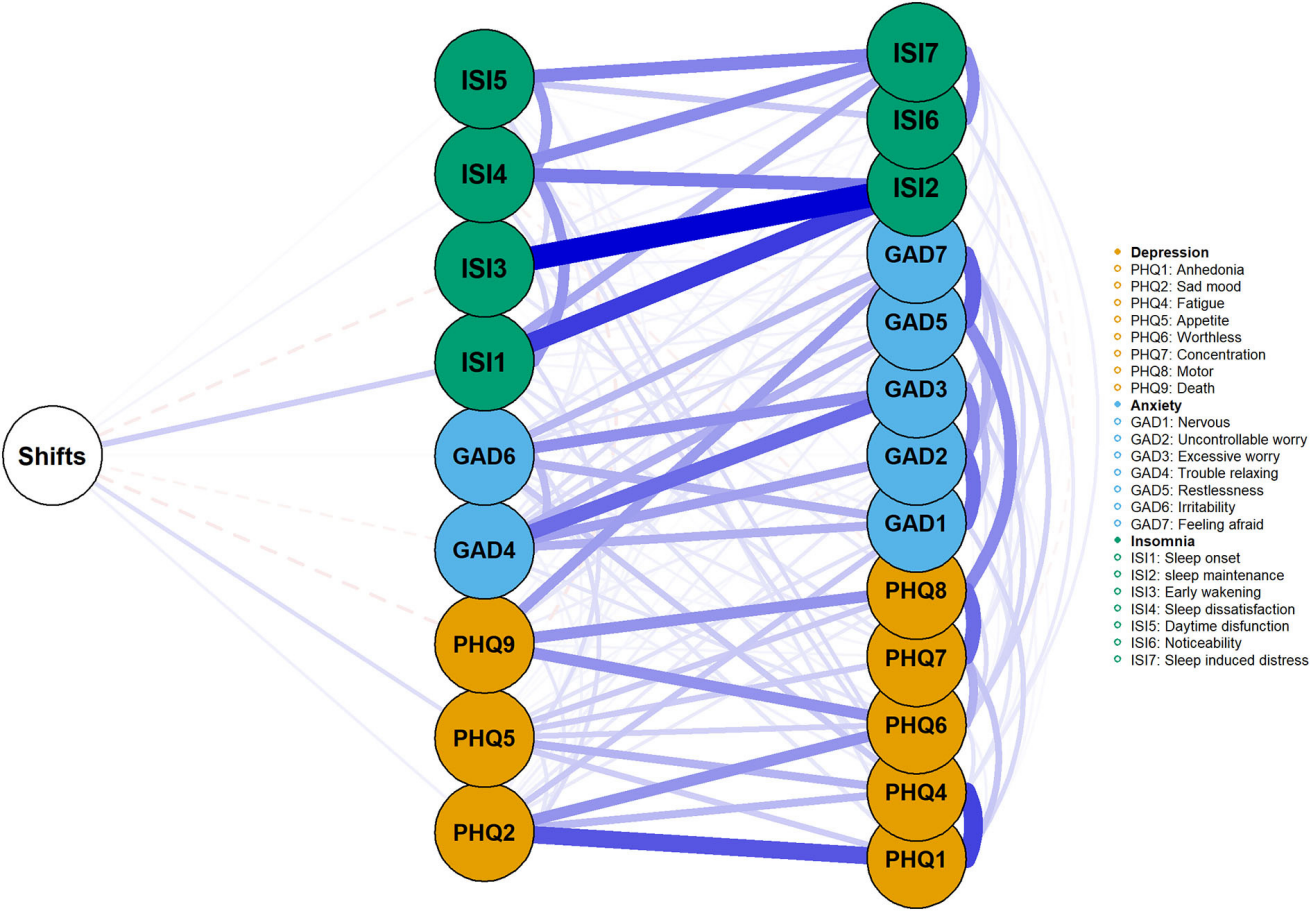
The impact of night shifts on insomnia, depression, and anxiety symptoms. The green, blue, and orange nodes represented insomnia, anxiety, and depression symptoms, respectively. The blue edges represented positive association, while the red represented the negative association. Thicker edges suggested stronger association.

We added monthly night shift frequency to the network (Figure 4). Having more night shifts was positively related to ISI1 (sleep onset), PHQ2 (sad mood), and PHQ5 (appetite). However, its associations with other symptoms were rather weak.

### Network comparison test

We compared insomnia–depression–anxiety network symptoms based on COVID-19 frontline experience (Supplementary Figure S3). We observed no differences in the global strength (*p* = 0.779) or network invariance (*p* = 0.479) between the two groups.

## Discussion

To our knowledge, this study was the first to assess the network structure of insomnia, depression, and anxiety symptoms in the nursing population. We identified several central symptoms (i.e., impaired sleep maintenance, trouble relaxing, and uncontrollable worry) and bridge symptoms (i.e., psychomotor agitation/retardation and restlessness). Fatigue and feeling afraid were both central and bridge symptoms. Night shifts were strongly associated with late sleep onset. COVID-19 frontline experience did not affect the network structure.

To date, only a few studies have assessed the comorbid of anxiety, depression, and insomnia at an item level (40–44). Despite the different study sample, sleep maintenance problem has been repeatedly reported to play an important part in the establishment of depressive, anxiety, and insomnia symptoms network (42–44), which were in line with our studies. Our work suggested sleep maintenance problems might be the network's trigger and driver. This hypothesis was supported by a retrospective study which found that approximately 66% of 456 insomnia patients first experienced sleep maintenance difficulties (45). Previous studies showed life stressors to have a stronger association with impaired sleep maintenance, compared with late sleep onset and early waking (46, 47). These studies might suggest that sleep maintenance problems are easily triggered by negative life events, and then induce other symptoms in the network.

We found that sleep disturbance symptoms showed higher strength than daytime dysfunction symptoms, contrasting Bai's study on mental health care workers at the pandemic's peak (39). This inconsistency might result from the different pandemic periods. Heavy workloads and serious consequences from medical errors during the day at the pandemic's peak may have led healthcare workers to focus more on daytime dysfunction than sleep disturbance in relation to insomnia. This finding highlights the need to track dynamic changes in insomnia among healthcare workers during the COVID-19 pandemic to provide targeted interventions.

Fatigue was defined as feeling tired and loss of energy (48). It emerged as both a central and bridge symptom within the network, implying it is an important clinical target. Fatigue's high centrality has been consistently reported in different populations, including college students, nursing students, and the general population, in the pandemic's late stage, indicating it might be the hallmark of depressive symptoms during this period (18–20, 40). Interestingly, fatigue has traditionally been recognized as a somatic symptom of depression, and a systemic review of the network of major depressive disorder suggested a robust community of fatigue, concentration loss, and psychomotor symptoms (49). However, we found fatigue had a stronger association with mood disturbances (anhedonia, sad mood, and feeling worthless) than somatic or cognitive symptoms. Within the depression community, PHQ4 (fatigue)–PHQ1 (anhedonia) was the strongest edge, while fatigue showed no association with concentration loss or psychomotor symptoms. This might suggest that fatigue in this sample was more likely to be psychological exhaustion rather than physical tiredness. Fatigue also showed positive associations with all insomnia symptoms except for noticeability and was most strongly associated with subjective sleep dissatisfaction and daytime dysfunction. Interestingly, the association between early waking and fatigue was rather weak.

In addition to fatigue, our findings suggested psychomotor symptoms, such as psychomotor agitation/retardation and restlessness, could trigger connections within the network. The interconnection between restlessness and psychomotor agitation/retardation was the network's most robust transdiagnostic edge. Other psychomotor symptoms, such as trouble relaxing, also showed high central strength. The high bridge centrality of psychomotor symptoms has been consistently validated in studies regarding the network of anxiety and depression during the COVID-19 pandemic (18, 20, 21, 50–52). This might reflect the impact of movement restrictions related to social distancing and lockdown policies on mental health during the pandemic, which requires focused attention (20). Sleep onset had the highest BEI among insomnia symptoms, suggesting it shares a close relationship with anxiety and depression symptoms. Our results support several longitudinal studies which found that sleep onset insomnia was a stronger predictor of depression than other insomnia subtypes (53, 54). Moreover, the residua of sleep onset insomnia were found to predict major depressive disorder relapse (55).

We also evaluated the impact of COVID-19 frontline experience and night shifts on mental health symptoms. We found no differences in the network between frontline and non-frontline nurses, which might have resulted from when data were collected. As the data were collected during the pandemic's remission period, the impact of direct exposure to COVID-19 patients on mental health might have disappeared. Several studies reported similar results to ours. Zhang et al. compared frontline and non-frontline nurses' mental health during the remission period. They found no difference in depression, anxiety, and insomnia prevalence between frontline and non-frontline nurses (56). Yu et al. described the network of depression and anxiety symptom network among Chinese clinicians and determined there was no difference in the symptom network between frontline and non-frontline clinicians.

Our study supported previous findings that night shifts were associated with insomnia and depression (57, 58). Particularly, night shifts were related to late sleep onset, rather than sleep maintenance or early wakening problems. Our results are in line with one previous study (59), which found significantly longer sleep latency in shift-work nurses. Moreover, we found night shifts were related to depressed mood and appetite change. These findings suggest the need to develop an optimal shift schedule and screen and intervene for late sleep onset insomnia and depression in nurses working night shifts.

This study has several implications for clinical practice and nursing management. First, our study demonstrated the long-lasting psychological harm of the pandemic on nurses during the remission period. Depression (59.6%), anxiety (47%), and insomnia (55.5%) symptoms showed higher prevalence than in studies during the pandemic's peak (60–63), suggesting the strong need to screen and intervene for depression, anxiety, and insomnia in this period. Second, our study identified several key symptoms, such as sleep maintenance, fatigue, psychomotor agitation/retardation, restlessness, and feeling afraid. They might play an important role in triggering and maintaining the depression-anxiety-insomnia network. Hence, it's necessary for nursing managers and policymakers to provide timely screening and targeted intervention for these specific symptoms, which might help early detect and reduce depression, anxiety, and insomnia in nurses. For example, a network intervention analysis revealed the effectiveness of behavior therapy on sleep maintenance (64), indicating it might be a promising treatment for insomnia among nurses. Physical activity might help reduce psychomotor symptoms and energy loss (65), which might be included in treatment interventions. Third, we found nurses with more night shifts were more prone to late sleep onset. Timely screening and prevention for late sleep onset insomnia are needed in this population. Cognitive behavioral therapy for insomnia has been demonstrated to be effective in treating insomnia and depression in shift workers (66, 67), which might help reduce insomnia in nurses working night shifts.

Our study had several limitations. First, owing to its cross-sectional design, causal relationships could not be identified. Second, as we did not collect baseline data, we could not provide dynamic trajectories for participants' mental well-being. Third, we used snowball sampling rather than random sampling, which might reduce the representativeness of our samples and lead to potential sampling bias. However, the reported prevalence of depression and anxiety and the demographic characteristics (age, gender, married status, and night shifts) were very close to those of one national cohort of Chinese nurses (*N* = 138, 279) in a similar period (68), suggesting such bias might be very small. Fourth, our study was conducted during the remission period of the pandemic and only included Chinese nurses. The work burden was lighter due to the control of the pandemic. Further studies are in need to verify our findings in different settings such as different periods of the pandemic and hospitals in other countries. In addition, we only assessed the frequency of the night shifts. Providing a more precise description of night shifts, such as frequency of consecutive shifts, length and intensity of night shifts, and shift patterns may help to better understand the relationship between night shifts, depression, anxiety, and insomnia in the nursing population. Fifth, mental distress was assessed *via* self-report questionnaires, rather than a standard diagnostic tool. Lastly, the use of the bootstrap procedure to assess the network stability might be another limitation of our study.

## Conclusion

Our study assessed the network structure of insomnia, anxiety, and depression symptoms among a large sample of nurses during the remission period of the COVID-19 pandemic. We found sleep maintenance was the central symptom, while fatigue, psychomotor agitation/retardation, restlessness, and feeling afraid were the bridge symptoms within the network. Night shifts were associated with a higher risk of depression, anxiety, and insomnia and exhibited a direct association with late sleep onset. These findings provided new insights into the symptom-symptom relationship of insomnia, depression, and anxiety and were valuable in preventing and treating the three common mental distresses in the nursing population.

## Data availability statement

The raw data supporting the conclusions of this article will be made available by the authors, without undue reservation.

## Ethics statement

The studies involving human participants were reviewed and approved by the Ethics Committee of the Second Xiangya Hospital of Central South University. The patients/participants provided their written informed consent to participate in this study.

## Author contributions

PP: conceptualization, software, and writing—original draft preparation and editing. ML: methodology and writing—review and editing. QWa: data curation and writing—review and editing. LL: writing—review and editing and validation. QC: design, supervision, and project administration. QWu: writing—review and editing, conceptualization, and methodology. All authors contributed to the article and approved the submitted version.

## Funding

This work was supported by the Emergency Response Special Project on the Novel Coronavirus Pneumonia of Hunan Provincial Scientific and Technological Department, China (Grant No. 2020SK3004 to QC).

## Conflict of interest

The authors declare that the research was conducted in the absence of any commercial or financial relationships that could be construed as a potential conflict of interest.

## Publisher's note

All claims expressed in this article are solely those of the authors and do not necessarily represent those of their affiliated organizations, or those of the publisher, the editors and the reviewers. Any product that may be evaluated in this article, or claim that may be made by its manufacturer, is not guaranteed or endorsed by the publisher.
