# Supplementary material for: Night shifts, insomnia, anxiety, and depression among Chinese nurses during the COVID-19 pandemic remission period: A network approach
Source: Front Public Health. 2022 Dec 5;10:1040298. doi: 10.3389/fpubh.2022.1040298 (PMC9760836; doi:10.3389/fpubh.2022.1040298)
Supplement: Supplementary file 1 [file Data_Sheet_1.docx]

Supplementary material for “Night shifts, insomnia, anxiety, and depression among Chinese nurses during the COVID-19 pandemic remission period: a network approach”

Table S1 Correlation matrix of PHQ9, GAD7, and ISI items

Table S2 Strength, Betweenness, Expected Influence, and Closeness of nodes

Figure S1 Estimation of edge difference by bootstrapped difference test

Figure S2 Estimation of node expected influence difference by bootstrapped difference test

Figure S3 Network structure of insomnia-depression-anxiety network in frontline and non-frontline nurses

Table S1 Correlation matrix of PHQ9, GAD7, and ISI items

|  | PHQ1 | PHQ2 | PHQ4 | PHQ5 | PHQ6 | PHQ7 | PHQ8 | PHQ9 | GAD1 | GAD2 | GAD3 | GAD4 | GAD5 | GAD6 | GAD7 | ISI1 | ISI2 | ISI3 | ISI4 | ISI5 | ISI6 | ISI7 |
| --- | --- | --- | --- | --- | --- | --- | --- | --- | --- | --- | --- | --- | --- | --- | --- | --- | --- | --- | --- | --- | --- | --- |
| PHQ1 | 0.000 | 0.296 | 0.328 | 0.086 | 0.020 | 0.097 | 0.004 | 0.000 | 0.067 | 0.012 | 0.008 | 0.000 | 0.000 | 0.041 | 0.000 | 0.000 | -0.002 | 0.000 | 0.000 | 0.044 | 0.000 | 0.000 |
| PHQ2 | 0.296 | 0.000 | 0.120 | 0.043 | 0.187 | 0.000 | 0.036 | 0.042 | 0.095 | 0.036 | 0.011 | 0.021 | 0.026 | 0.060 | 0.012 | 0.004 | 0.000 | 0.000 | 0.000 | 0.000 | 0.000 | 0.023 |
| PHQ4 | 0.328 | 0.120 | 0.000 | 0.130 | 0.046 | 0.000 | 0.000 | 0.000 | 0.043 | 0.005 | 0.021 | 0.025 | 0.000 | 0.092 | 0.000 | 0.047 | 0.036 | 0.002 | 0.063 | 0.062 | 0.000 | 0.029 |
| PHQ5 | 0.086 | 0.043 | 0.130 | 0.000 | 0.080 | 0.089 | 0.090 | 0.000 | 0.050 | 0.016 | 0.000 | 0.011 | 0.010 | 0.019 | 0.004 | 0.062 | 0.016 | 0.021 | 0.000 | 0.018 | 0.000 | 0.000 |
| PHQ6 | 0.020 | 0.187 | 0.046 | 0.080 | 0.000 | 0.134 | 0.063 | 0.188 | 0.010 | 0.051 | 0.072 | 0.047 | 0.000 | 0.020 | 0.075 | 0.000 | 0.000 | 0.000 | 0.000 | 0.000 | -0.031 | 0.000 |
| PHQ7 | 0.097 | 0.000 | 0.000 | 0.089 | 0.134 | 0.000 | 0.257 | 0.018 | 0.000 | 0.022 | 0.034 | 0.014 | 0.034 | 0.035 | 0.000 | 0.044 | 0.000 | 0.000 | 0.000 | 0.000 | 0.000 | 0.004 |
| PHQ8 | 0.004 | 0.036 | 0.000 | 0.090 | 0.063 | 0.257 | 0.000 | 0.186 | 0.009 | 0.031 | 0.000 | 0.000 | 0.203 | 0.000 | 0.063 | 0.000 | 0.002 | 0.041 | -0.038 | 0.024 | 0.053 | 0.000 |
| PHQ9 | 0.000 | 0.042 | 0.000 | 0.000 | 0.188 | 0.018 | 0.186 | 0.000 | 0.000 | 0.029 | 0.000 | 0.000 | 0.045 | 0.000 | 0.168 | 0.004 | 0.002 | 0.000 | -0.043 | -0.020 | 0.026 | 0.006 |
| GAD1 | 0.067 | 0.095 | 0.043 | 0.050 | 0.010 | 0.000 | 0.009 | 0.000 | 0.000 | 0.251 | 0.119 | 0.135 | 0.024 | 0.135 | 0.019 | 0.025 | 0.000 | 0.000 | 0.000 | 0.015 | 0.000 | 0.003 |
| GAD2 | 0.012 | 0.036 | 0.005 | 0.016 | 0.051 | 0.022 | 0.031 | 0.029 | 0.251 | 0.000 | 0.211 | 0.162 | 0.135 | 0.005 | 0.112 | 0.000 | 0.001 | 0.000 | 0.000 | 0.000 | 0.000 | 0.000 |
| GAD3 | 0.008 | 0.011 | 0.021 | 0.000 | 0.072 | 0.034 | 0.000 | 0.000 | 0.119 | 0.211 | 0.000 | 0.255 | 0.000 | 0.191 | 0.078 | 0.014 | 0.014 | 0.013 | 0.014 | 0.000 | -0.024 | 0.000 |
| GAD4 | 0.000 | 0.021 | 0.025 | 0.011 | 0.047 | 0.014 | 0.000 | 0.000 | 0.135 | 0.162 | 0.255 | 0.000 | 0.138 | 0.113 | 0.058 | 0.010 | 0.057 | 0.000 | 0.009 | 0.000 | -0.006 | 0.031 |
| GAD5 | 0.000 | 0.026 | 0.000 | 0.010 | 0.000 | 0.034 | 0.203 | 0.045 | 0.024 | 0.135 | 0.000 | 0.138 | 0.000 | 0.050 | 0.270 | 0.026 | 0.000 | 0.016 | -0.031 | 0.000 | 0.034 | 0.000 |
| GAD6 | 0.041 | 0.060 | 0.092 | 0.019 | 0.020 | 0.035 | 0.000 | 0.000 | 0.135 | 0.005 | 0.191 | 0.113 | 0.050 | 0.000 | 0.124 | 0.000 | 0.039 | 0.000 | 0.034 | 0.023 | -0.008 | 0.000 |
| GAD7 | 0.000 | 0.012 | 0.000 | 0.004 | 0.075 | 0.000 | 0.063 | 0.168 | 0.019 | 0.112 | 0.078 | 0.058 | 0.270 | 0.124 | 0.000 | 0.020 | 0.000 | 0.023 | -0.009 | 0.000 | 0.013 | 0.024 |
| ISI1 | 0.000 | 0.004 | 0.047 | 0.062 | 0.000 | 0.044 | 0.000 | 0.004 | 0.025 | 0.000 | 0.014 | 0.010 | 0.026 | 0.000 | 0.020 | 0.000 | 0.341 | 0.069 | 0.186 | 0.000 | 0.009 | 0.151 |
| ISI2 | -0.002 | 0.000 | 0.036 | 0.016 | 0.000 | 0.000 | 0.002 | 0.002 | 0.000 | 0.001 | 0.014 | 0.057 | 0.000 | 0.039 | 0.000 | 0.341 | 0.000 | 0.445 | 0.226 | 0.005 | 0.015 | 0.042 |
| ISI3 | 0.000 | 0.000 | 0.002 | 0.021 | 0.000 | 0.000 | 0.041 | 0.000 | 0.000 | 0.000 | 0.013 | 0.000 | 0.016 | 0.000 | 0.023 | 0.069 | 0.445 | 0.000 | 0.072 | 0.000 | 0.025 | 0.030 |
| ISI4 | 0.000 | 0.000 | 0.063 | 0.000 | 0.000 | 0.000 | -0.038 | -0.043 | 0.000 | 0.000 | 0.014 | 0.009 | -0.031 | 0.034 | -0.009 | 0.186 | 0.226 | 0.072 | 0.000 | 0.174 | 0.000 | 0.203 |
| ISI5 | 0.044 | 0.000 | 0.062 | 0.018 | 0.000 | 0.000 | 0.024 | -0.020 | 0.015 | 0.000 | 0.000 | 0.000 | 0.000 | 0.023 | 0.000 | 0.000 | 0.005 | 0.000 | 0.174 | 0.000 | 0.100 | 0.213 |
| ISI6 | 0.000 | 0.000 | 0.000 | 0.000 | -0.031 | 0.000 | 0.053 | 0.026 | 0.000 | 0.000 | -0.024 | -0.006 | 0.034 | -0.008 | 0.013 | 0.009 | 0.015 | 0.025 | 0.000 | 0.100 | 0.000 | 0.202 |
| ISI7 | 0.000 | 0.023 | 0.029 | 0.000 | 0.000 | 0.004 | 0.000 | 0.006 | 0.003 | 0.000 | 0.000 | 0.031 | 0.000 | 0.000 | 0.024 | 0.151 | 0.042 | 0.030 | 0.203 | 0.213 | 0.202 | 0.000 |

Table S2 Strength, Betweenness, Expected Influence, and Closeness of nodes

| node | Item content | Betweenness | Closeness | Strength | Expected Influence |
| --- | --- | --- | --- | --- | --- |
| PHQ1 | Anhedonia | 0.51 | 1.05 | 0.25 | 0.41 |
| PHQ2 | Sad mood | 0.00 | 0.51 | 0.29 | 0.47 |
| PHQ4 | Fatigue | 1.27 | 1.34 | 0.51 | 0.66 |
| PHQ5 | Appetite | -0.94 | 0.27 | -1.29 | -0.96 |
| PHQ6 | Worthless | -0.51 | 0.16 | 0.37 | 0.21 |
| PHQ7 | Concentration | -0.77 | 0.65 | -1.07 | -0.76 |
| PHQ8 | Motor | 1.36 | 1.50 | 0.83 | 0.55 |
| PHQ9 | Death | -0.34 | 0.66 | -1.09 | -1.46 |
| GAD1 | Nervous | -0.77 | -0.12 | 0.21 | 0.40 |
| GAD2 | Uncontrollable worry | -0.68 | 0.24 | 0.69 | 0.83 |
| GAD3 | Excessive worry | -0.77 | 0.15 | 0.69 | 0.58 |
| GAD4 | Trouble relaxing | 1.19 | 1.03 | 0.77 | 0.84 |
| GAD5 | Restlessness | 1.10 | 0.78 | 0.47 | 0.30 |
| GAD6 | Irritability | -0.68 | 0.18 | 0.17 | 0.27 |
| GAD7 | Feeling afraid | -0.17 | 0.16 | 0.65 | 0.70 |
| ISI1 | Sleep onset | -0.34 | -0.82 | 0.28 | 0.46 |
| ISI2 | Sleep maintenance | 1.95 | -0.48 | 1.67 | 1.69 |
| ISI3 | Early wakening | -1.11 | -1.33 | -1.21 | -0.89 |
| ISI4 | Sleep dissatisfaction | 1.87 | -0.50 | 0.83 | -0.34 |
| ISI5 | Daytime disfunction | -1.20 | -1.71 | -1.57 | -1.43 |
| ISI6 | Noticeability | -0.68 | -2.09 | -2.46 | -2.76 |
| ISI7 | Sleep induced distress | -0.26 | -1.61 | 0.00 | 0.20 |

Figure S1 Estimation of edge difference by bootstrapped difference test


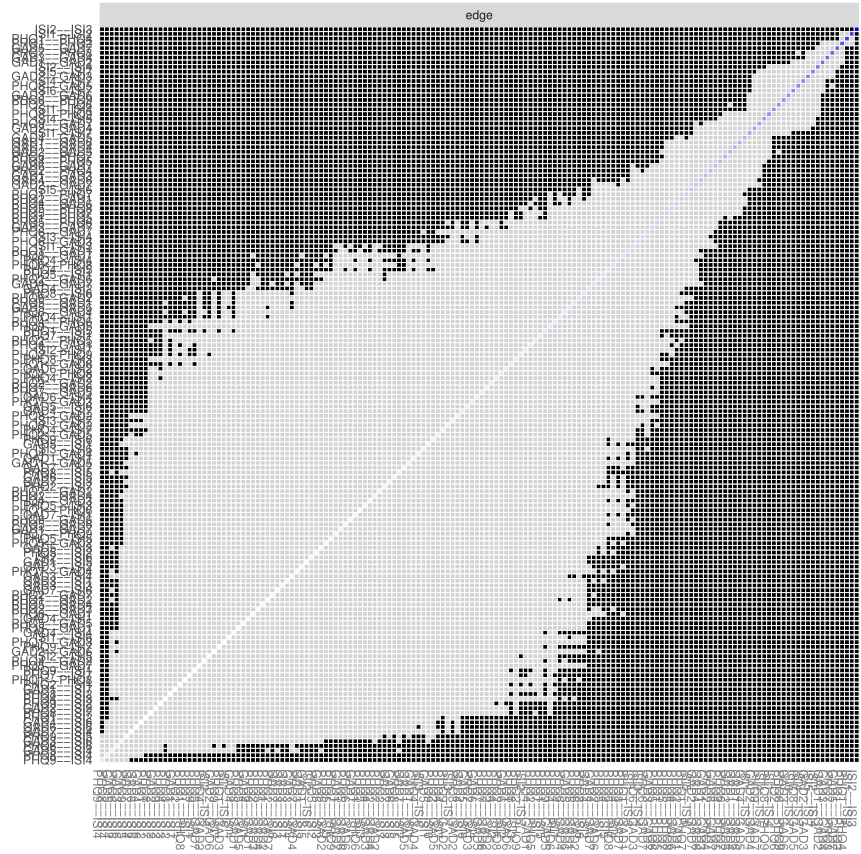


Figure S1. Bootstrapped difference tests between edges. Gray boxes indicate edges that do not significantly differ from one another. Black boxes represent edges that differ significantly from one another (α = 0.05).

Figure S2 Estimation of node expected influence difference by bootstrapped difference test


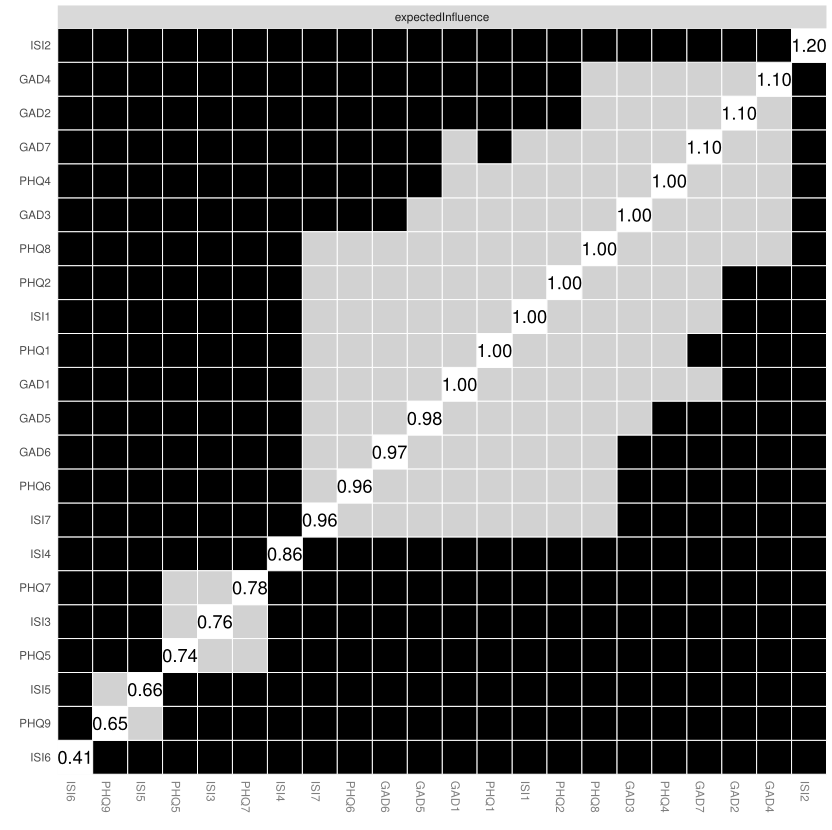


Figure S2. Bootstrapped difference tests between node expected influence of factors. Gray boxes indicate nodes that do not significantly differ from one another. Black boxes represent nodes that differ significantly from one another (α = 0.05). White boxes show the values of node expected influence.

Figure S3 Network structure of insomnia-depression-anxiety network in frontline and non-frontline nurses


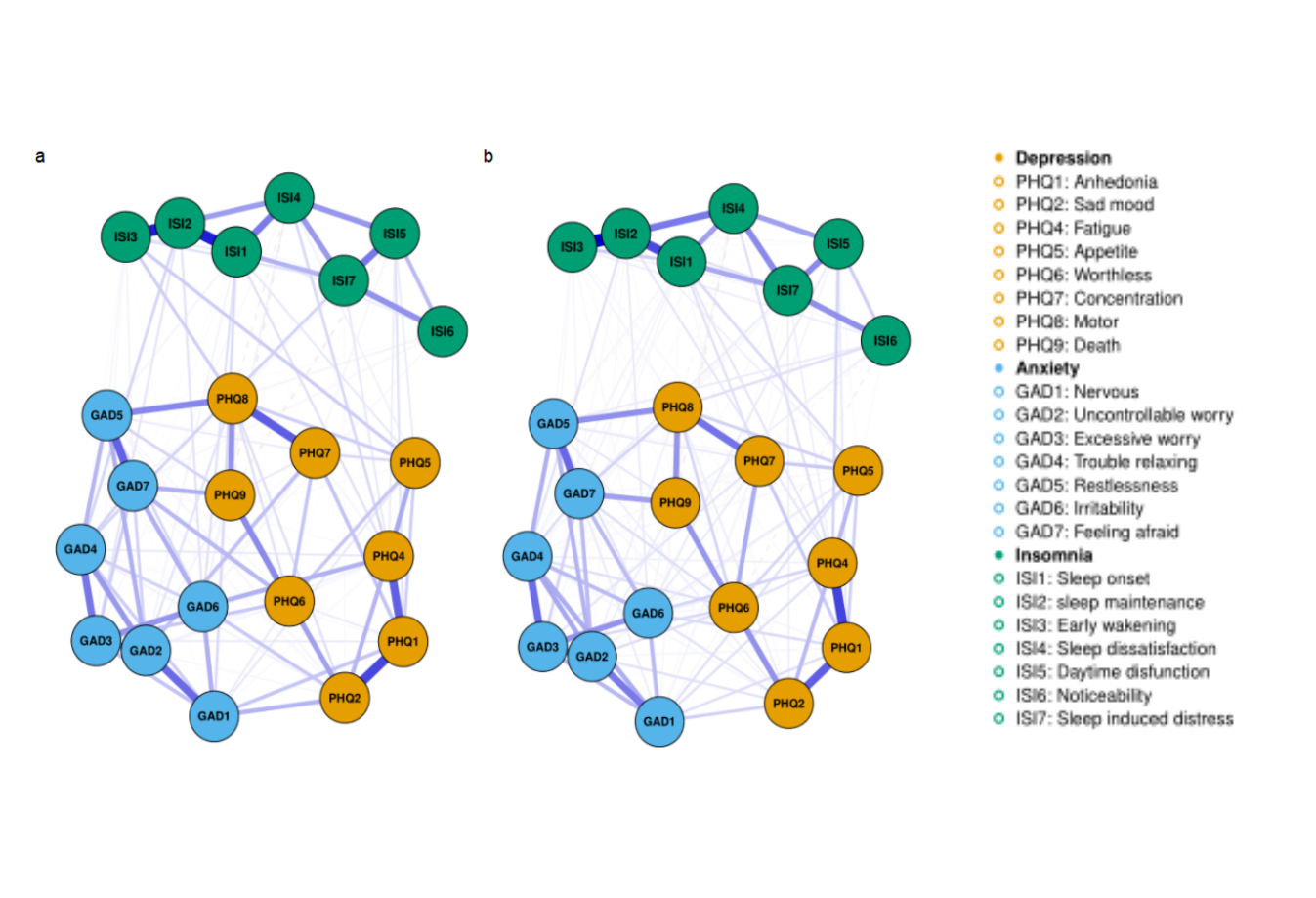


Figure S3. S3a Network structure of insomnia, anxiety, depression symptoms in frontline nurses. S4a Network structure of insomnia, anxiety, depression symptoms in non-frontline nurses.
